# Primary effusion lymphoma in a human immunodeficiency virus-negative patient with unexpected unusual complications: a case report

**DOI:** 10.1186/s13256-019-2221-6

**Published:** 2019-09-23

**Authors:** Liliana Fernández-Trujillo, John E. Bolaños, Mauricio Velásquez, Carlos García, Luz F. Sua

**Affiliations:** 1 Department of Internal Medicine, Pulmonology Service, Interventional Pulmonology, Fundación Valle del Lili, Avenida Simón Bolívar, Cra 98 No. 18-49, Fundación Valle del Lili. Tower 6, 4th Floor, Office 446, 760032 Cali, Colombia; 2 Faculty of Health Sciences, Universidad Icesi, Cali, Colombia; 3 Department of Surgery, Thoracic Surgery Service, Fundación Valle del Lili, Cali, Colombia; 4 Department of Radiology, Fundación Valle del Lili , Cali, Colombia; 5 Department of Pathology and Laboratory Medicine, Fundación Valle del Lili, Cali, Colombia

**Keywords:** Diffuse large B-cell lymphoma, Primary effusion lymphoma, Human herpes virus-8, Pseudoaneurysms, Heart rupture

## Abstract

**Background:**

Primary effusion lymphoma is a rare, high-grade non-Hodgkin’s lymphoma that usually occurs in immunosuppressed or human immunodeficiency virus-positive individuals in advanced stages of the disease. However, primary effusion lymphoma occasionally affects immunocompetent patients who are infected with human herpes virus type 8 or Epstein-Barr virus. This disease manifests with liquid collections in cavities, producing constitutional symptoms; fever; weight loss; and symptoms related to extrinsic compression, such as dyspnea or abdominal discomfort. Diagnosis is confirmed with cytological or tissue evaluation showing large, multinucleated lymphoid cells with positive specific markers for the disease, such as CD45 and markers related to viral infections, when present. There is no standard treatment for primary effusion lymphoma, but several chemotherapy protocols are recommended, usually with poor results.

**Case presentation:**

We present a case of an adult human immunodeficiency virus-negative Hispanic origin woman with primary effusion lymphoma with pleuritic, pericardial, and peritoneal compromise who also had unusual complications during a diagnostic procedure: the accidental rupture of the left ventricle and the development of a secondary left ventricular pseudoaneurysm. We describe the clinical, radiological, and laboratory characteristics as well as the outcome of this case.

**Conclusions:**

Primary effusion lymphoma is a very rare entity that represents 4% of non-Hodgkin’s lymphoma cases associated with human immunodeficiency virus and 0.1% to 1% of all lymphomas in patients with another type of immunodeficiency in regions where human herpes virus type 8 is not endemic. This reported case is an unusual presentation of primary effusion lymphoma because it occurred in an immunocompetent human immunodeficiency virus-negative adult woman without the presence of Kaposi’s sarcoma or Castleman’s disease and for whom the clinical course after chemotherapy was successful. However, the rupture of the free wall of the left ventricle is a very rare catastrophic event that usually occurs after myocardial infarction. Left ventricle free wall rupture rarely goes unnoticed, but when it occurs, it leads to the development of a ventricular pseudoaneurysm in which the rupture is contained by the pericardium with an organized thrombus and an adjacent hematoma.

## Introduction

Primary effusion lymphoma (PEL) is a rare, high-grade non-Hodgkin’s lymphoma [1–3]. PEL occurs most commonly in human immunodeficiency virus (HIV)-positive young men in advanced stages of the disease and in individuals with acquired immunodeficiency syndrome (AIDS) or immunosuppressed HIV-negative individuals as a result of a solid organ transplant, neoplastic disease, autoimmune disease, or cirrhosis, among other pathologies [4–7]. However, occasionally, PEL can affect immunocompetent patients, usually elderly individuals, infected with human herpes virus type 8 (HHV-8) or Epstein-Barr virus (EBV) [3, 7–12]. Thus, PEL occurs in association with these viruses in both immunocompetent and immunosuppressed individuals [12].

This neoplasm manifests with liquid lymphomatous collections located in the pleural, pericardial, or peritoneal cavity, without extracavitary masses, lymphadenopathies, or enlarged lymph nodes [1, 2, 8]. The symptoms are secondary to the effusion, malignancy in this case, producing a mass effect and extrinsic compression of the adjacent organs. Consequently, in pericardial and pleural presentations, the cardinal symptom is dyspnea [13].

The diagnosis is confirmed by cytological evaluation of the fluid and/or biopsy of the compromised membrane. Large, multinucleated or multilobed lymphoid cells with prominent nucleoli, basophilic cytoplasm with clear small vacuoles, and high mitotic activity are observed. These cells show a CD45^+^ immunophenotype and are usually negative for B-cell markers, such as CD19, CD20, and CD79a. Surface and cytoplasmic immunoglobulins are absent, as is B-cell lymphoma 6 protein (BCL6). Activation markers, plasma cell markers, and associated markers that do not define the lineage are positive, such as human leukocyte antigen (HLA)-DR, CD30, CD38, and VS38c, a monoclonal antibody that recognizes a rough endoplasmic reticulum (rER) intracellular antigen termed “cytoskeleton-linking membrane protein 63.” rER is typically found in viable tumor cells and is abundant in plasma cells, CD138, and epithelial membrane antigen (EMA) [14, 15]. These cells usually do not express T/natural killer cell line markers. When viral infection is present, the nucleus is positive for HHV-8 associated with the latent nuclear antigen protein (ORF73), which is very useful to establish the diagnosis [16].

Approximately half of the patients have a preexisting diagnosis of Kaposi’s sarcoma or multicentric Castleman’s disease [17, 18]. Although there is no standard treatment for PEL, combined chemotherapy, called “EPOCH,” which consists of etoposide phosphate, prednisone, vincristine sulfate, cyclophosphamide, and doxorubicin hydrochloride, is recommended [13, 19]. However, despite treatment, the prognosis is poor, given its high resistance to chemotherapy, with an average survival of less than 6 months [13, 20–22]. Few patients have a response to chemotherapy and/or immune modulation.

We present a case of an HIV-negative adult woman with PEL with pleural, pericardial, and peritoneal compromise who consulted for presenting constitutional symptoms and prolonged fever before the diagnosis was confirmed and had unusual complications during a diagnostic procedure: accidental rupture of the left ventricle and the development of a secondary left ventricular pseudoaneurysm. We describe the clinical, radiological, and laboratory characteristics and the outcome of this case.

## Case presentation

A 60-year-old Hispanic origin woman with no relevant history consulted for a 2-month evolution of constitutional symptoms, malaise, low-grade fever, chills, and dyspnea that had progressed from medium effort to rest over the course of a few days before consultation. She worked as a secretary in a home appliances shop. She had no history of hypertension, diabetes, or asthma and no cigarette, alcohol, or drug use or exposure to particulate matter or chemical substances. She had had two cesarean sections, the last one 30 years prior. Her father died of coronary disease at 80 years of age, and her mother died of a cerebral ischemic event at 75 years of age. She did not refer to cough, chest or joint pain, hemoptysis, rash, gastrointestinal symptoms, syncope, or neurological compromise, and she denied leaving her city of residence during the last 6 months, having pets, or taking specific medications.

Her physical examination on admission revealed the following: blood pressure (BP) of 82/57 mmHg, heart rate of 81 beats/minute, respiratory rate of 22 breaths/minute, and body temperature of 38.3 °C. The patient was alert, afebrile, hydrated, with pale mucous membranes, and without signs of respiratory distress. Her neck was without lymphadenopathy or jugular engorgement. Symmetric thorax with normal expansion and rhythmic heart without galloping, friction rub, or murmurs were observed. She had diminished breath sounds in both lung bases, in the abdomen, with no hepatomegaly or splenomegaly, no edema in the lower limbs, and no skin lesions. The result of her neurological evaluation was normal.

**Fig. 1:**
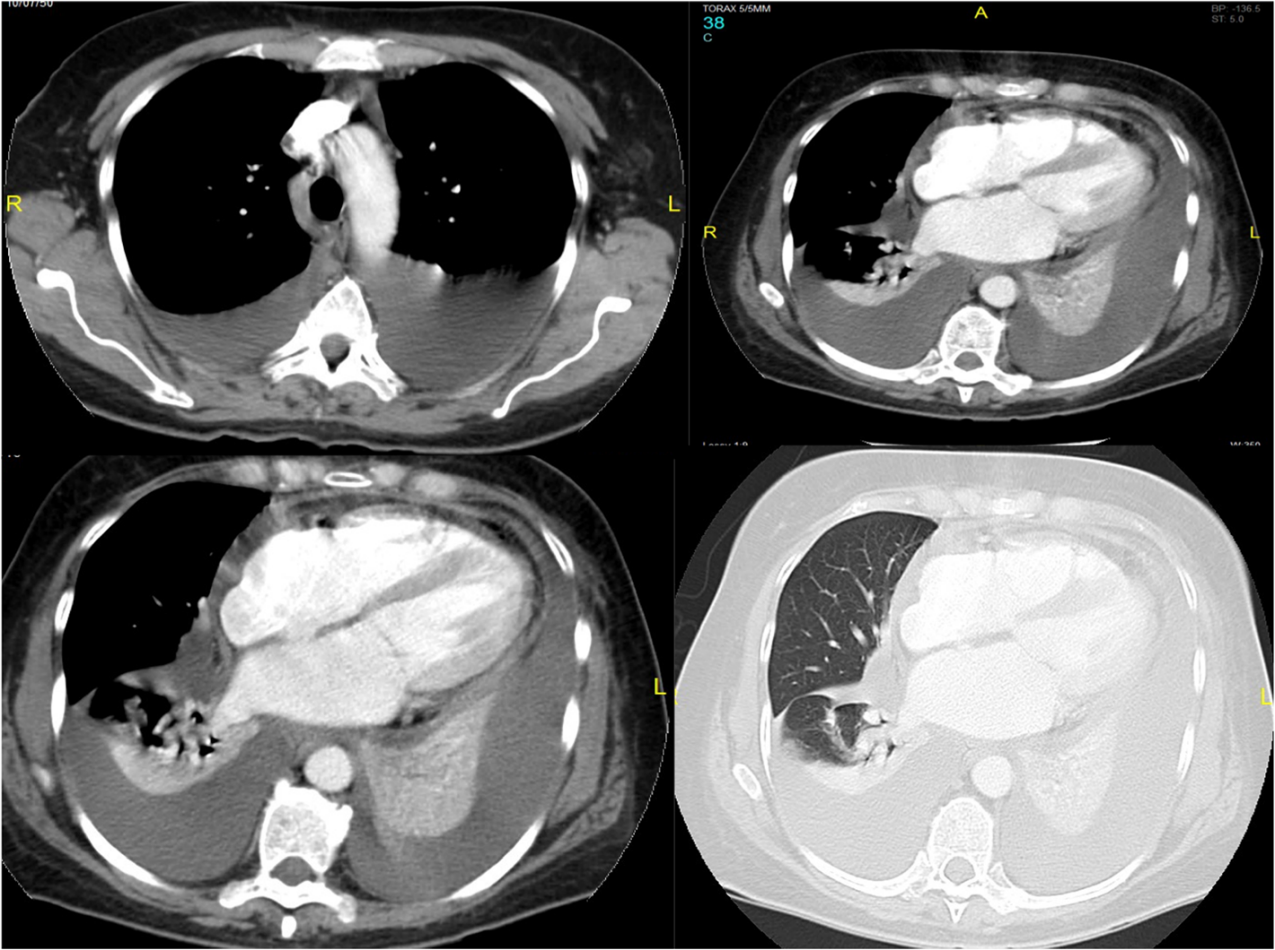
Different aspects of the thoracic computed tomographic scan showing bilateral pleural effusion, thickening of the pericardium, and pericardial effusion, without evidence of enlarged lymph nodes or pulmonary infiltrates. *R* right side, *L* left side

Laboratory tests revealed the following values: hemoglobin 9 mg/dl, leukocytes count 10,200 per microliter, neutrophils 75%, lymphocytes 15%, platelets in normal range, creatinine 0.9 mg/dl, lactate dehydrogenase (LDH) 350 mg/dl, liver test results within normal parameters, and normal urinalysis. Her rheumatoid factor, antinuclear antibodies, extractable nuclear antigens, perinuclear and cytoplasmic antineutrophil cytoplasmic antibodies, and VDRL (i.e., Venereal Disease Research Laboratory) test results were negative. Her test results for hepatitis and HIV were negative. Her C-reactive protein was 30 mg/ml, thyroid-stimulating hormone 15 μIU/ml, and B-type natriuretic peptide 1553 pg/ml. An echocardiogram revealed a left ventricular ejection fraction of 60%. Moderate pericardial effusion that could explain the presence of BP 82/57 mmHg as an incipient sign of cardiac tamponade was observed. A thoracic computed tomographic (CT) scan revealed free bilateral pleural effusion, pericardial thickening, and pericardial effusion without enlarged lymph nodes (Fig. 1). Thoracentesis was performed, which produced a straw-colored fluid with a predominance of lymphocytes, and the result of cytology was negative for malignancy. No flow cytometry was performed. The results of direct examination and cultures for bacteria, mycobacteria, and fungi were negative, as were those for blood cultures, urine cultures, and GeneXpert (Cepheid, Sunnyvale, CA, USA) evaluation for tuberculosis. The patient’s presentation evolved with persistent fever, so treatment for hypothyroidism was initiated. The effusion persisted, and for determination of a lymphoproliferative disease, the patient underwent bone marrow and hepatic biopsies, the results of which were inconclusive. A thoracoscopy was subsequently performed for biopsies of the pleura and pericardium and a new study of the fluid. During this procedure, when the pericardium was approached, an accidental rupture of the left ventricle occurred, which, owing to the local inflammatory process, was tightly adhered to the pericardium.

This rupture was immediately recognized, and the ventricle was sutured during the same procedure with the assistance of the cardiovascular surgery group without instant additional complications. Before the surgical procedure, the patient received antibiotic prophylaxis with cefazolin 2 g intravenously, and after the rupture she received cefepime 2 g intravenously every 8 hours and vancomycin 15 mg/kg intravenously for 7 days.

**Fig. 2:**
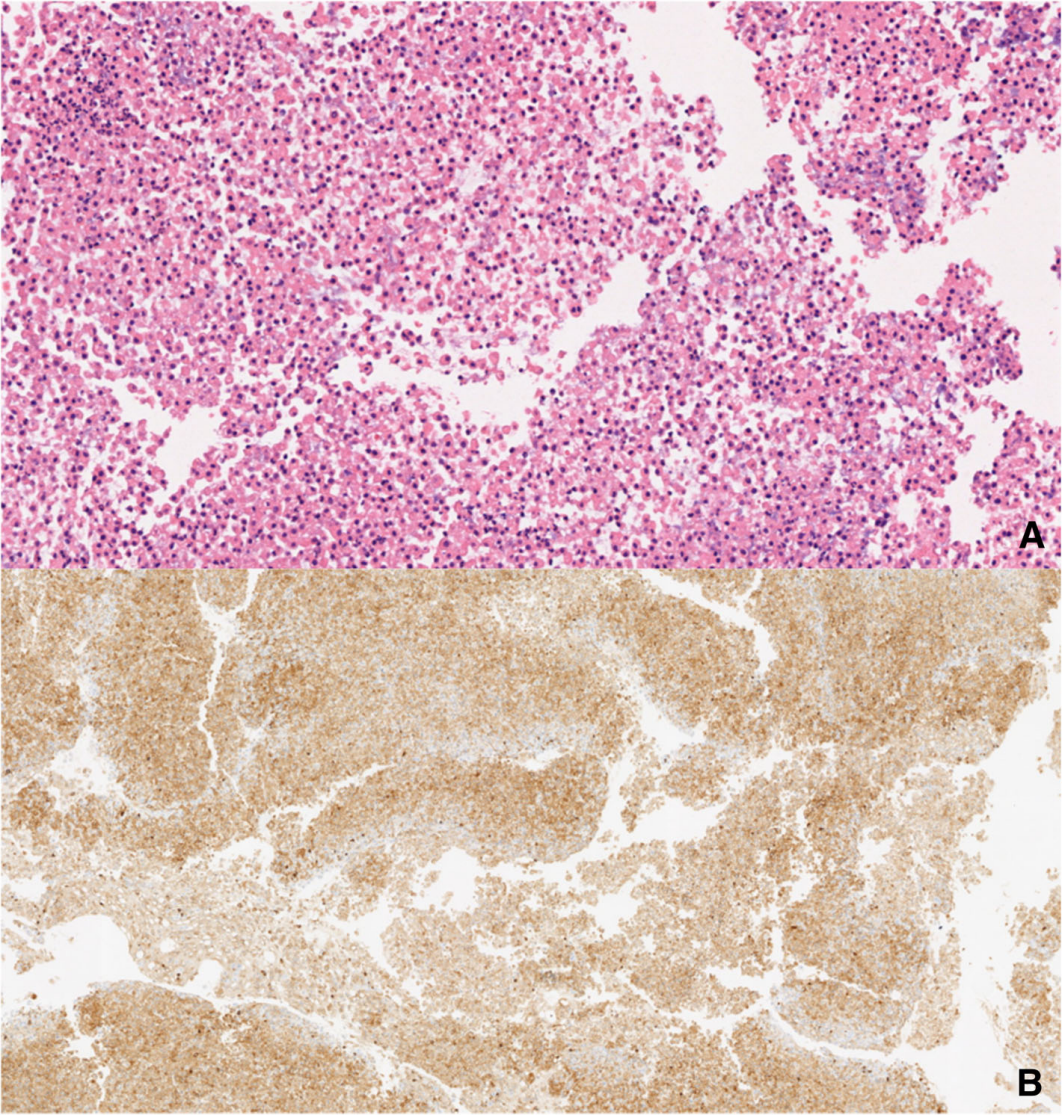
**a** Neoplastic lymphocytes obtained from pleural effusion (H&E stain). **b** IHC showing expression of CD45 (leukocyte common antigen), confirming the hematolymphoid cell line

Biopsy of the serosal membranes showed pleura and pericardium with collagenization and fibrosis. Flow cytometry of the pleural fluid revealed a positive cell population for CD45, CD38, and HLA-DR that was negative for B and T lymphoid cell line markers. In the cell block, a lymphoid population was identified, which by IHC expressed CD45, CD38, EMA, myeloperoxidase, and HHV-8 and was negative for CD20, CD79a, CD3, CD5, CD56, BCL2, BCL6, CD117, HLA-DR, CD15, CD30, and EBV latent membrane protein 1, confirming a diagnosis of primary serous lymphoma associated with HHV-8 (Fig. 2a, b).

**Fig. 3:**
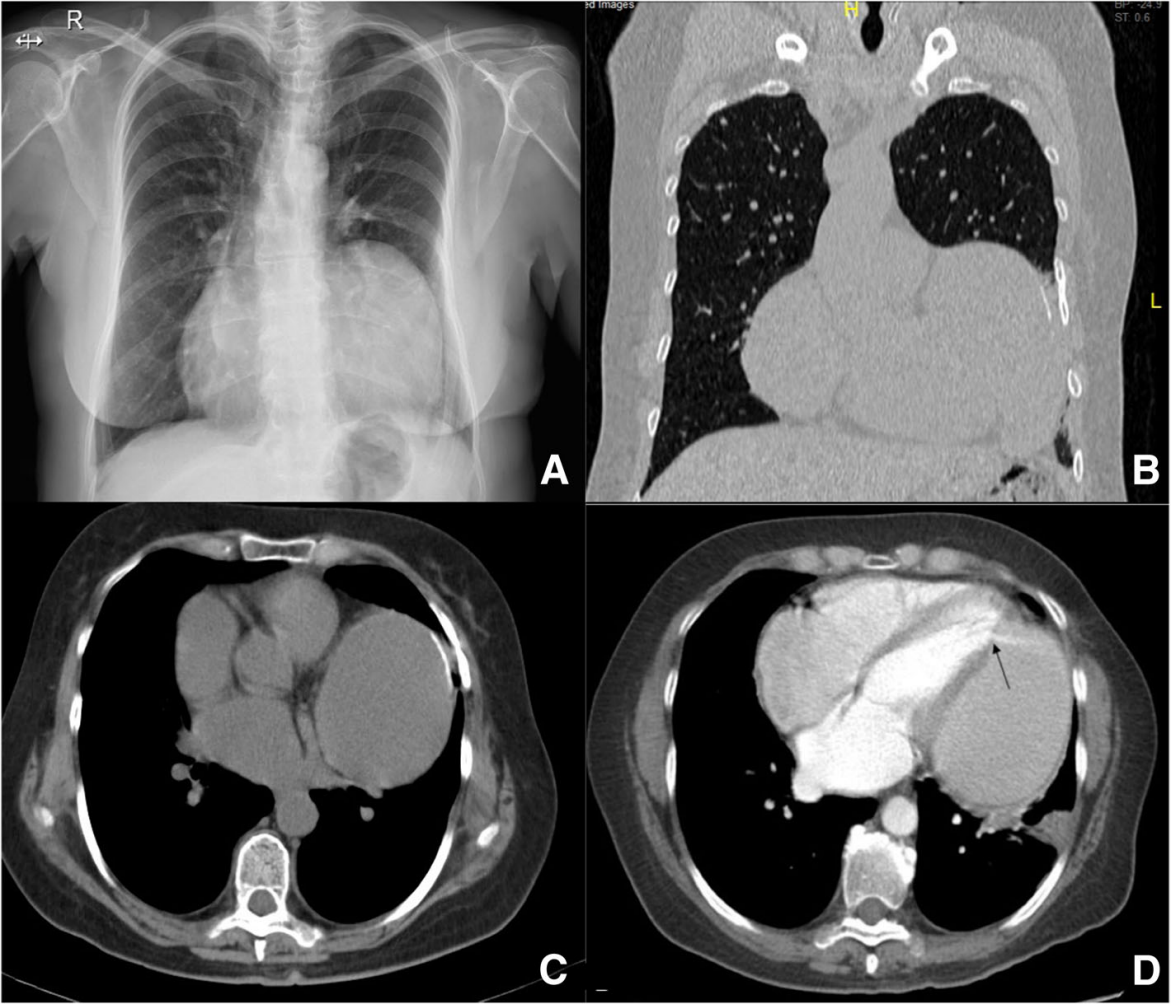
**a** Chest x-ray showing ballooning of the left cardiac silhouette, without pulmonary infiltrates or pleural effusion. **b** Sagittal scan of the left ventricular lesion. **c** Thoracic computed tomographic (CT) scan: appearance of the ventricular pseudoaneurysm with thin pericardial wall. **d** Thoracic CT-scan showing the leakage of contrast medium within the cavity of the ventricular pseudoaneurysm, and this corresponds exactly to the black arrow. *R* right side, *L* left side

Treatment was initiated with chemotherapy, cyclophosphamide, vincristine, and prednisone, eight cycles in total. The initial clinical picture improved, with effervescence of the fever and constitutional symptoms. The patient was discharged to continue outpatient management by the hemato-oncology department and has remained without evidence of disease recurrence for 2 years. Regarding the cardiac lesion, the patient had follow-up with ultrasound and CT that evidenced preservation of ventricular function and the appearance of a large ventricular pseudoaneurysm that has remained stable over time (Fig. 3a–d). The patient currently has a good functional status without chest pain or dyspnea and continues being seen as an outpatient by the cardiology department. She denied accepting the option of surgery to correct the cardiac injury that was offered after evaluation of the case in conjunction with cardiovascular surgery. She has been seen by the cardiology team twice each year for two consecutive years.

## Discussion

The reported case is an unusual presentation of PEL because it occurred in an immunocompetent HIV-negative adult woman without the presence of Kaposi’s sarcoma or Castleman’s disease and for whom the clinical course after chemotherapy has been successful. This patient also had an unusual complication related to thoracic diagnostic procedures: rupture of the free wall of the left ventricle, which subsequently evolved to the long-term development of a ventricular pseudoaneurysm, a situation that put this patient’s life at risk.

PEL is a very rare entity that represents 4% of non-Hodgkin’s lymphomas associated with HIV and 0.1% to 1% of all lymphomas in patients with another type of immunodeficiency in regions where HHV-8 is not endemic [23, 24]. PEL usually occurs more frequently in young men with HIV or in individuals with severe immunodeficiency who are infected with HHV-8, and in many cases, there is coinfection with EBV [25].

There is a report of two cases of PEL in HIV-negative older women in an area with a high prevalence of HHV-8 infection, specifically in the Mediterranean region, but with a previous diagnosis of Kaposi’s sarcoma [26]. In Latin America, the incidence is unknown, particularly in the HIV-negative population.

HHV-8 encodes more than ten homologous cell proliferation and antiapoptotic signaling genes and is the probable etiological agent of Kaposi’s sarcoma. DNA sequences of HHV-8 have been found in lesions of Kaposi’s sarcoma, PEL, and multicentric Castleman’s disease by polymerase chain reaction and *in situ* hybridization. Latent nuclear antigen (LNA-1, LNA, LANA-1), also known as “ORF73,” is a 222 or 234 kD protein that is consistently expressed in cells infected with HHV-8 [27], which was positive by IHC in our patient’s case.

In general, on one hand, the prognosis is related to the extension of the disease, extranodal involvement, LDH concentration, bone marrow involvement, age, number of CD4 lymphocytes in peripheral blood, functional status, and previous diagnosis of AIDS. On the other hand, the rupture of the free wall of the left ventricle is a very rare catastrophic event that usually occurs after myocardial infarction. Rupture of the left ventricle rarely goes unnoticed, but when it occurs, it leads to the development of a ventricular pseudoaneurysm [28, 29], in which the rupture is contained by the pericardium, with an organized thrombus and an adjacent hematoma. The true ventricular aneurysm, unlike the pseudoaneurysm, retains a thin ventricular wall. In our patient’s case, there was an accidental injury of the myocardium with initial repair that evolved to the formation of the pseudoaneurysm, as shown in the figures. These lesions have a high risk of rupture, and surgical repair was recommended [30–34].

The clinical course of our patient demonstrates a good response to oncological treatment without the recurrence of lymphoproliferative disease during 2 years of follow-up and without spontaneous rupture of the ventricular pseudoaneurysm, a possibility at any moment after the establishment of this exotic clinical condition.

## Conclusion

We report a rare case of PEL that occurred in an immunocompetent patient who presented initially with latent manifestations and extensive involvement of the serosa; the presence of HHV-8 was also detected. The patient had a good long-term evolution with oncological treatment. A high index of suspicion is important in the evaluation of patients with serosal involvement without clear or evident diagnosis by means of cytology or flow cytometry of lymphoproliferative cells suggesting the compromise by this type of lymphoma. Our patient also had an unusual complication related to thoracic diagnostic procedures: the rupture of the free wall of the left ventricle, which evolved to the long-term development of a ventricular pseudoaneurysm, posing a high risk for future complications, such as spontaneous rupture of the pericardium that contains the pseudoaneurysm. In this sense, the recommendation is surgical correction when it can be performed.

## Data Availability

All data and materials are available for sharing if needed.

## Abbreviations

AIDS: Acquired immunodeficiency syndrome
BCL6: B-cell lymphoma 6 protein
BP: Blood pressure
CD45: Cluster of differentiation 45
EBV: Epstein-Barr virus
EMA: Epithelial membrane antigen
EPOCH: Etoposide phosphate, prednisone, vincristine sulfate, cyclophosphamide, and doxorubicin hydrochloride
HHV-8: Human herpes virus type 8
HIV: Human immunodeficiency virus
HLA: Human leukocyte antigen
LANA: Latent nuclear antigen
LDH: Lactate dehydrogenase
PEL: Primary effusion lymphoma
rER: Rough endoplasmic reticulum
